# Improvements in the production of purified M13 bacteriophage bio-nanoparticle

**DOI:** 10.1038/s41598-020-75205-3

**Published:** 2020-10-29

**Authors:** Paolo Passaretti, Inam Khan, Timothy R. Dafforn, Pola Goldberg Oppenheimer

**Affiliations:** 1 Institute of Cancer and Genomic Sciences, University of Birmingham, Birmingham, B15 2TT UK; 2 School of Metallurgy and Materials, University of Birmingham, Birmingham, B15 2TT UK; 3 School of Biosciences, University of Birmingham, Birmingham, B15 2TT UK; 4 School of Chemical Engineering, University of Birmingham, Birmingham, B15 2TT UK

**Keywords:** Isolation, separation and purification, Biological techniques, Nanobiotechnology

## Abstract

M13 bacteriophage is a well-established versatile nano-building block, which can be employed to produce novel self-assembled functional materials and devices. Sufficient production and scalability of the M13, often require a large quantity of the virus and thus, improved propagation methods characterised by high capacity and degree of purity are essential. Currently, the ‘gold-standard’ is represented by infecting *Escherichia coli* cultures, followed by precipitation with polyethylene glycol (PEG). However, this is considerably flawed by the accumulation of contaminant PEG inside the freshly produced stocks, potentially hampering the reactivity of the individual M13 filaments. Our study demonstrates the effectiveness of implementing an isoelectric precipitation procedure to reduce the residual PEG along with FT-IR spectroscopy as a rapid, convenient and effective analytic validation method to detect the presence of this contaminant in freshly prepared M13 stocks.

## Introduction

M13 is a filamentous virus which infects bacteria (bacteriophage) and in particular, the *Escherichia* *coli* (*E.* *coli*) strains showing the F-*pilus*^1^. It measures approximately  1 µm in length and  6 nm in width. It is essentially comprised of a circular single-stranded DNA (ssDNA) molecule surrounded by a protein capsid mostly made of the major coat protein (PVIII) and other four minor coat proteins (PIII, PVI, PVII and PIX)^2,3^. There are about 2700 copies of PVIII assembled along the body of the phage and at the two extremities, there are approximately five copies of PIII and PVI on a side and PVII and PIX to the other^2,3^.

Since its discovery, M13 has been extensively studied to both characterise its components as well as to understand its biological role and mechanism of replication^2^. Furthermore, due to the discovery of restriction enzymes and the development of more advanced molecular biology technics for the modification of the DNA, M13 has become an intriguing nanotool for novel applications^4–6^. For instance, the ‘phage display’^7^, which is the capability of M13 to display fused peptides and proteins on its minor and major coat proteins, laid the groundwork for the study of protein–protein, peptide–protein interactions^8^, for the development of molecular probes for cell imaging^9,10^ and the detection of microorganisms^11^. Moreover, since in the last two decades M13 have been employed for the fabrication of advanced nanostructured functional material and devices, there is a growing demand for purified phage^12–15^, requiring alternative methods of propagation, purification and quantification which are crucial for the scalability of the M13 production.

Bulk production of highly purified M13 virions is crucial for many of the application which requires the virus to interact with other components at a molecular level such as for instance, the development of bioassays, molecular probes and novel nanostructured materials^14,16–23^.

Although many methods have been employed for the propagation and purification of M13, *i.e.,* the infection of *E. coli* in fed-batch cultures^24^ and continuous fermenters^25–27^, as well as purification via gel-electrophoresis^28,29^, desalting spin column^30^, ion exchange (IEC)^31–33^ and size-exclusion chromatography (SEC)^32,34^, the most common method remains the propagation in *E. coli* batch cultures followed by precipitation with polyethylene glycol (PEG)^35^.

Although these methods do not require particularly sophisticated equipment, the PEG precipitation is inherently disadvantageous, producing M13 stocks with a significantly high content of residual PEG^36^. This can be reduced introducing an additional purification step, i.e., the isoelectric precipitation^36^. This method consists of lowering the pH around the isoelectric point (IEP) of the M13^37^ followed by producing the flocculation of the viral particles dispersed in solution and eventually, spinning down to obtain a pellet. Subsequently, the latter can be resuspended in PBS, deionised water (DIW) or any other buffer of choice for specific applications.

In addition to the production of viral stocks containing residues of PEG potentially hampering the interaction of M13 with other components^36^, the contaminant PEG itself is not easily detectable with the conventional UV–Vis spectrophotometric analysis, often employed for the quantification of the phage stocks. This is because the signal from the most intense absorption peak of PEG is overlapping with the *π* → *π*^*∗*^ transitions in the peptide bonds (*λ* = 180–230 nm)^38,39^.

The interaction of PEG with proteins have been widely studied over time. PEG can interact via a variety of non-covalent interactions such as for instance, the hydrophobic attraction and multipoint van der Waals contacts due to its amphiphilicity^40^. Moreover, the external hydroxyl groups of PEG and the interior chain groups, interact either weakly or strongly with many other chemical groups commonly present in proteins such as carboxylates, amides, hydroxyls, aliphatic and aromatic carbons^41^. It is also known that high molecular weight PEGs (MW > 5 kDa) could affect the microenvironment and the conformation of proteins and enzymes, leading to a change of their activity^40–42^. Therefore, to minimise the side effects caused by the presence of PEG, it is necessary to minimise its presence in freshly produced M13 stocks.

Here we propose an alternative procedure for the propagation, purification and quantification of the M13. In particular, after the batch propagation, the purification via PEG (M13_PEG_) precipitation includes an additional step of isoelectric precipitation at pH 4.0 to remove the contaminant PEG left during the M13_IEP_ step. Furthermore, we employ FT-IR spectroscopy to study the effectiveness of isoelectric precipitation in reducing the amount of contaminant PEG in the freshly produced M13 stocks in a rapid and reliable manner.

This work will be beneficial to physical, chemical, and biological science communities interested in propagating and purify high-quality M13 stocks. Moreover, this will lay the platform for improvements in the reactivity of the M13′s surface, otherwise hampered by the contaminant PEG for a breadth of applications in bio-nanotechnology and novel nanomaterials.

## Materials and methods

### M13 propagation and purification

**Figure 1:**
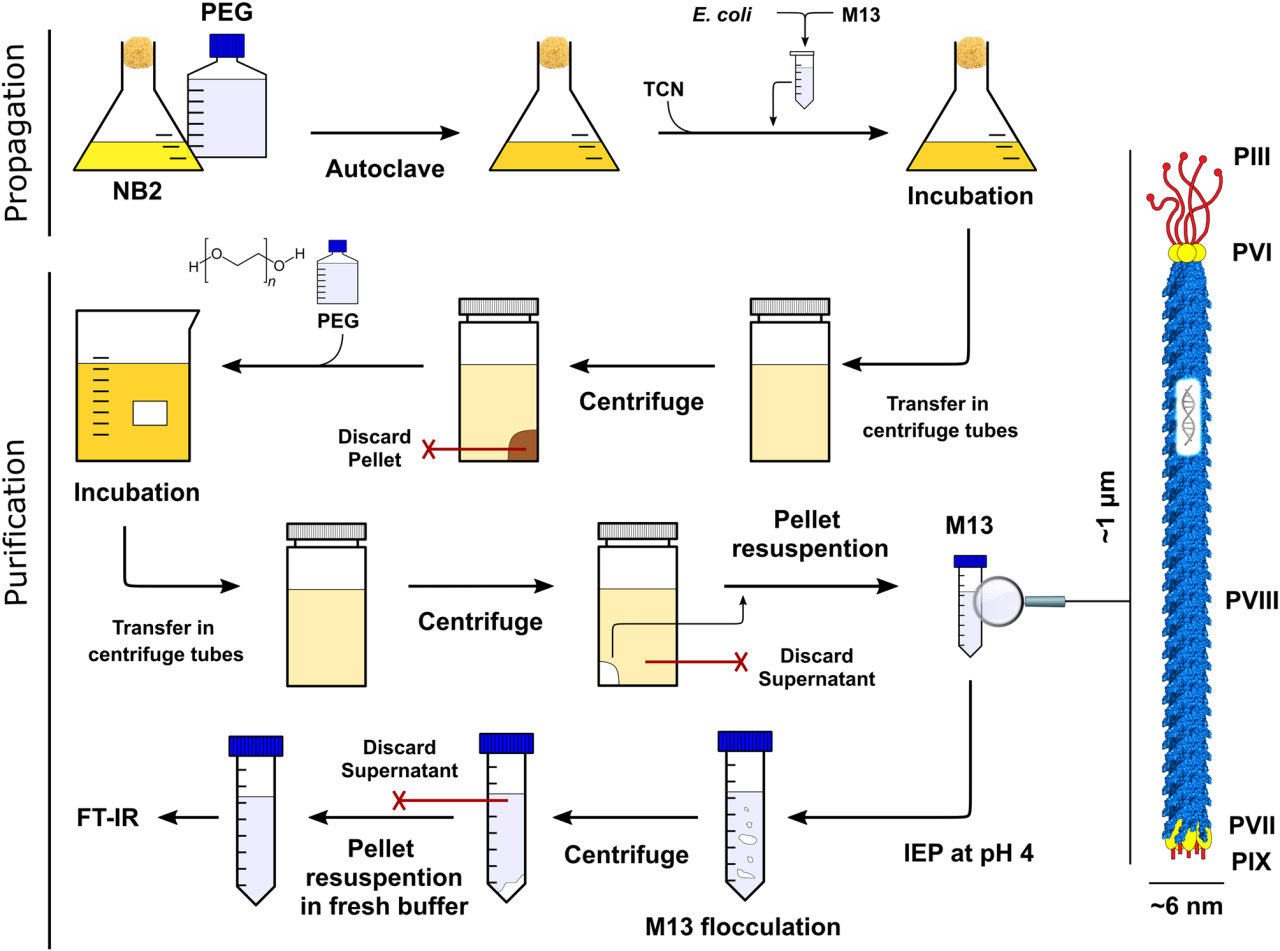
Schematic of the overall M13 propagation, purification and subsequent FT-IR analysis. The propagation of M13 is performed via infecting batch cultures of *E. coli.* Subsequently, after pelleting and removing the bacteria (brown pellet), the growing media is centrifuged in the presence of PEG and the pellet is collected (white pellet). Once resuspended in a buffer, the dispersed M13 is precipitated again by lowering the pH using HCl and centrifuging. Finally, the pellet is resuspended in a buffer and analysed via the FT-IR. The figure was created using Inkscape 1.0.1.

M13 bacteriophage (M13KE) was purchased from New England Biolabs as double-stranded DNA (dsDNA) and transferred into One Shot TOP10F´ Chemically Competent *E. coli* (Thermo Fisher Scientific) through a heat shock. It was subsequently self-propagated in batch cultures using the *E. coli* strain (Fig. 1). For this purpose, 2 L of Nutrient Broth No.2 (Thermo Fisher Scientific Oxoid) was autoclaved. Once cooled down to room temperature, tetracycline was added to a final concentration of 5 µg mL^−1^. Subsequently, M13 and *E. coli* at 37 °C, 150 rpm for 24 h. After the propagation, the culture was centrifuged twice at 8000 rpm (Beckman Coulter JA 10, RCF = 11,295.1 *g*) and the pellet containing the *E. coli* was discarded. The supernatant containing M13 was subsequently incubated with PEG 6000 (Sigma-Aldrich) and stirred for 90 min at 4 °C. Finally, the supernatant/PEG solution was centrifuged at 10,000 rpm (Beckman Coulter JA 10, RCF = 17,648.6 *g*) and the supernatant was discarded.

The obtained white pellet was resuspended in DIW. Subsequently, the pH of the solution was lowered to pH 4 using 5 M HCl, provoking the flocculation of M13.

The flocculated sample was then split into small microtube and centrifuged for 5 min at 15,000 rpm (SciSpin MICRO by SciQuip, RCF = 15,100 *g*) and the supernatant was discarded. The obtained white pellet consisting of M13 was resuspended in DIW and centrifuged again (5 min at 15,000 rpm) to remove any insoluble fraction. Finally, the concentration of M13 was determined with a UV–Vis spectrophotometer (CaryWinUV by Agilent Technologies), measuring the absorbance at 269 nm and using an extinction coefficient of *ε* = 3.84 ± 0.06 cm^2^ mg^−1^^37,39^.

### UV–Vis spectrophotometry

All samples were analysed in a 0.1 cm light path quartz cuvette (Aireka QG10001) using a UV–Vis spectrophotometer (Agilent Cary 60 UV–Vis). The background signal was measured accordingly using the same buffer in which the samples were dispersed.

### Fourier-transform infrared spectroscopy (FT-IR)

FT-IR spectrometer (Nicolet 6700 Thermo Fisher Scientific) was employed to study the fingerprint spectra of PEG and the M13. In particular, FT-IR was crucial to detect PEG contamination, consequent to the purification process. Samples were prepared by grinding 200 mg of pre-dried KBr together with 2 mg of sample (PEG, M13_PEG_ and M13_IEP_). Subsequently, the mortared samples were transferred into a 13 mm diameter compression mold and compacted under a pressure of 10 Tons. Transparent disks were obtained, ready to be analysed with the FT-IR spectrometer in a transmission mode. Before analysing the samples, a blank disk containing only KBr was prepared and used to obtain the background spectrum. The spectral region between 400 and 4000 cm^−1^ was measured with a resolution of 4 cm^−1^ and 100 scans to obtain the final spectra.

### Software

OriginLab pro 2019 was used for data analysis and to plot UV–Vis and FT-IR spectra.

## Results and discussion

**Figure 2:**
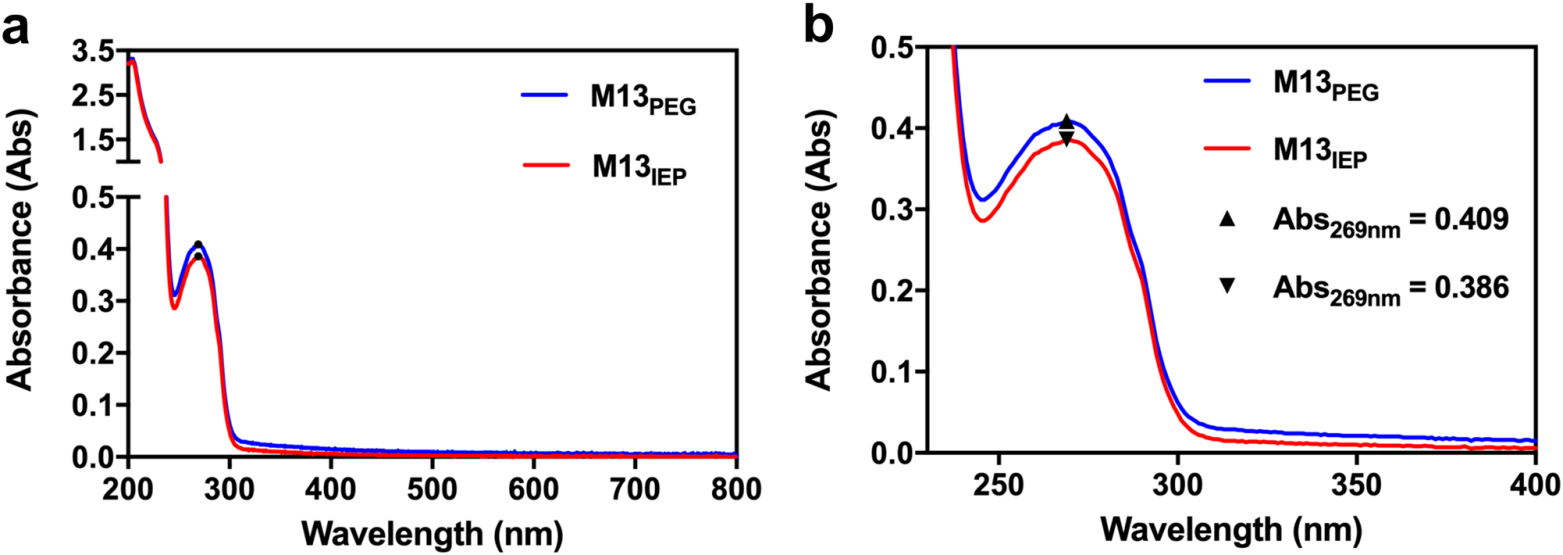
Representative UV–Vis spectra of the M13_PEG_ and the M13_IEP_. (**a**) UV–Vis spectra of M13 purified via PEG precipitation (M13_PEG_) and PEG/isoelectric precipitations M13_IEP_. (**b**) Zoomed-in UV–Vis spectra of the previous spectra (**a**), focusing on the range between 250–400 nm.

The UV–Vis spectra of the purified M13 using PEG (M13_PEG_) were compared to the ones acquired after further isoelectric precipitation (M13_IEP_). Since viruses are essentially made of protein and nucleic acids, the UV–Vis spectrum of M13 was found to be very similar to the combined spectrum of individual DNA and proteins (Fig. 2)^43^. DNA absorbs light between 150–300 nm and is showing two main peaks. The absorption below 180 nm is attributed to the deoxyribose while the one between 180–300 nm is due to the nitrogenous bases^44^. On the other hand, proteins absorb light in the range of 180–300 nm. The range of 180–230 nm is almost entirely attributed to the *π* → *π*^*∗*^ transitions in the peptide bonds. The absorption in the range of 230–300 nm is due to the aromatic side-chains of tryptophan (Trp), tyrosine (Tyr), and phenylalanine (Phe) residues, with a further weak contribution from the disulphide bonds near 260 nm^45–48^. The UV–Vis quantification of DNA and proteins is calculated using the absorption at 260 and 280 nm, respectively. Therefore, the presence of a peak at 269 nm and a higher absorption in the region below 240 nm in the UV–Vis spectrum of M13 (Fig. 2) is in correspondence with the previous reports^49^.

The comparison between the UV–Vis spectra of M13_PEG_ and M13_IEP_ shows a reduction in the overall signal of the M13_IEP_. The absorbance value of the M13 has decreased from 0.409 to 0.386 absorbance units, most probably due to the two additional centrifugation cycles needed for the isoelectric precipitation process. The reduced pH values during the isoelectric precipitation can damage the protein capsid of M13, destroying the viral particles, which precipitated in the reaction tube. However, this accounts for just 5.6% of the initial phage concentration. Furthermore, it is important to note that there are no detectable absorbance peaks between 320–800 nm, indicating that there are no other detectable components in the sample.

FT-IR was employed for the further and more detailed characterisation of the M13’s purity. Therefore, we focussed on the detailed analysis of the characteristic FT-IR fingerprint of the PEG and M13.

Although the purification of M13 using PEG is one of the most commonly used methods, the resultant purified phages are often contaminated with a correlating quantity of PEG, which could, in turn, impact phage’s further interactions during various self-assembly and functionalisation procedures in bio-nano engineering while reducing the overall quality of the purified product^50^. FT-IR is a suitable method to examine such contaminations as well as to evaluate further interactions in the fabrication of supramolecular structures containing M13^51,52^.

**Figure 3:**
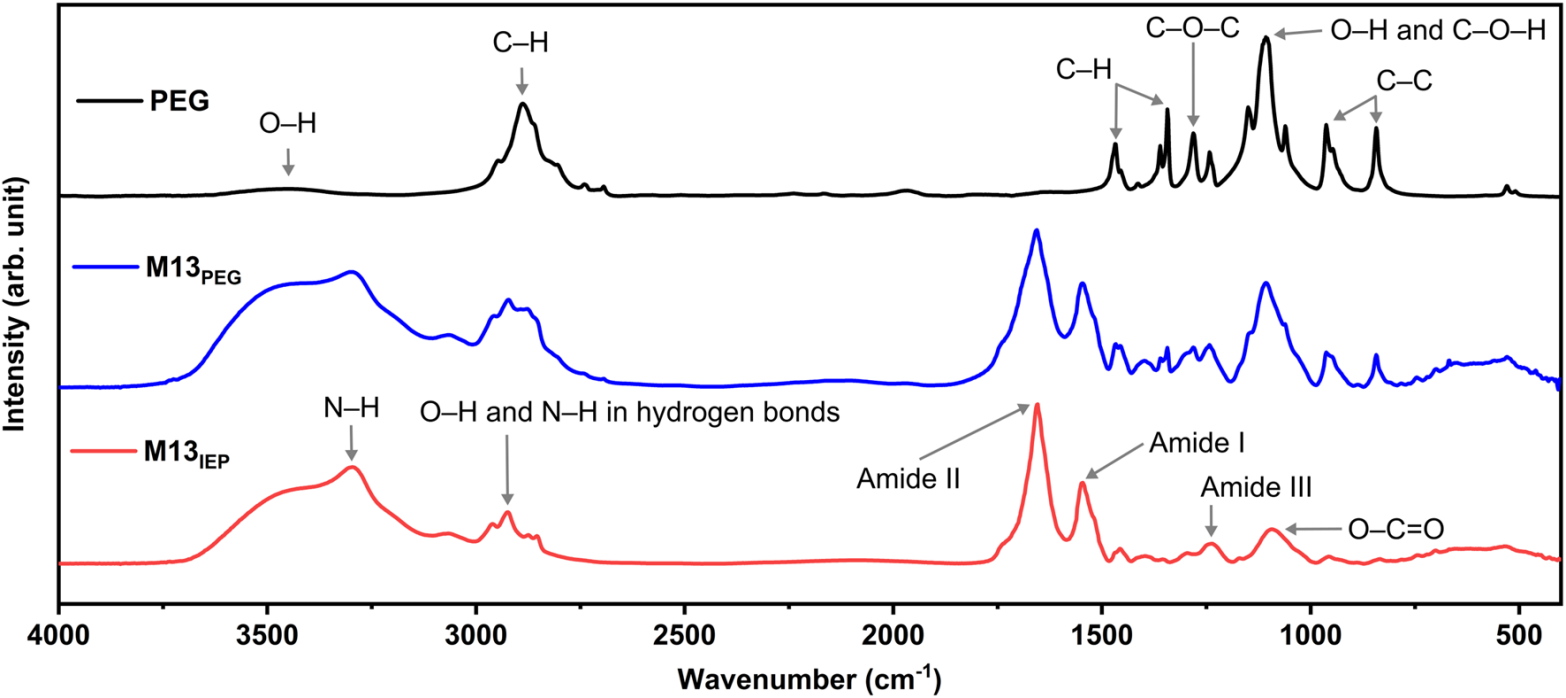
Representative FT-IR spectra of M13_PEG_ and M13_IEP_. The arrows in the spectra of PEG (black), M13 purified via PEG precipitation (M13_PEG_) (blue) and M13 after both, PEG and isoelectric precipitations (M13_IEP_) (red) indicate the most characteristic peaks of PEG and M13. All spectra are normalised to the maximum absorbance value.

Representative FT-IR spectra of PEG, M13_PEG_ and M13_IEP_ are shown in Fig. 3. M13_IEP_ was obtained after isoelectric precipitation of M13_PEG_ to investigate the effectiveness of this procedure on reducing the presence of contaminant PEG. The obtained FT-IR spectral assignments of specific functional groups of PEG and M13 are summarised in Table 1 and ESI Table S1–3^50,53–57^.

**Table 1:** Functional group assignments of PEG and M13.

Wavelength (cm^−1^)	PEG	M13	References
3600–3400	O–H aliphatic stretching	O–H aliphatic stretching	^50,53–57^
3296	–	N–H stretching (secondary amine)	
3000–2800	C–H stretching	C–H stretching	
1655	–	Amide I band: Amide plane C=O stretching	
1545	–	Amide II band: amide plane N–H bending	
1467–1456	C–H bending	C–H bending	
1395	–	Symmetric C–H_3_ bending	
1360–1350	C–H deforming	C–H deforming	
1342	C–H bending	–	
1296	–	C–N stretching	
1281	O–H and C–O–H stretching	–	
1243	C–O–C asymmetric stretching	–	
1238	–	Amide III band: C–N stretching and N–H in-plane bending	
1170–1150	C–O–H stretching	C–C and C–O–H stretching	
1108	O–H and C–O–H stretching	–	
1093	––	O–C=O stretching	
1060	C–O stretching	–	
962–843	C–C skeletal vibration	C–C skeletal vibration	

The samples were synthesised and analysed according to the procedures described in the ‘Materials and Methods’ section, and the obtained spectra were normalised and fitted for the corresponding peak assignments (Fig. 3). For the polyether PEG compound with the chemical formula of H–(O–CH_2_–CH_2_)_n_–OH, the average spectrum shows a characteristic broad absorption peak centred at ~ 3400 cm^−1^, assigned to the stretching vibration bands of hydroxyl groups (O–H). The triplet splitting peaks between 3000–2800 cm^−1^ is a feature of the C–H stretching vibration bands. The peak centred at ~ 1108 cm^−1^ with a triplet splitting pattern is commonly found in PEG, corresponding to the combination of O–H and C–O–H vibrational stretching bands.

M13_PEG_ spectrum shows a greater number of peaks compared to PEG (Fig. 3). However, certain similarities of the two spectral fingerprints indicate the presence of PEG such as for instance, the peaks centred at ~ 1108 cm^−1^ and the peaks corresponding to the C–C skeletal vibrational bands below 1000 cm^−1^.

The spectrum of M13_IEP_ was found to be similar to the M13_PEG_, however, with a notable reduction in the intensity of the peaks attributed to PEG (Fig. 3 and Table 1).

The spectrum of PEG, on the other hand, reveals a presence of distinctive peaks including 843, 947 and 962 cm^−1^, corresponding to carbon’s skeletal vibrations. M13_PEG_ and M13_IEP_ exhibit a few additional distinctive peaks in the range between 1000–1900 cm^−1^, corresponding to several bonds involving carbon i.e., C–O stretching of aliphatic ethers, O–H and C–O–H stretching and alkyl aryl ether C–O stretching as well as the amide band I, II and III (Table 1). The broad absorbance peak at ~ 3400 cm^−1^ is attributed to the O–H stretching whilst the peak at 3296 cm^−1^ is representative of the secondary amine (N–H) groups.

The comparison of the FT-IR peaks of PEG, M13_PEG_ and M13_IEP_ highlights the differences between the three samples (Fig. 3). The amide bands I and II respectively at 1545 and 1655 cm^−1^, together with the peak at ~ 3296 cm^−1^, are exclusive characteristics of M13 only. On the other hand, the strong and sharp peaks between the 800–1500 cm^−1^ can be associated with PEG, even though the chemical groups to which they correspond, are also present in the DNA and the constituting proteins of the M13.

**Figure 4:**
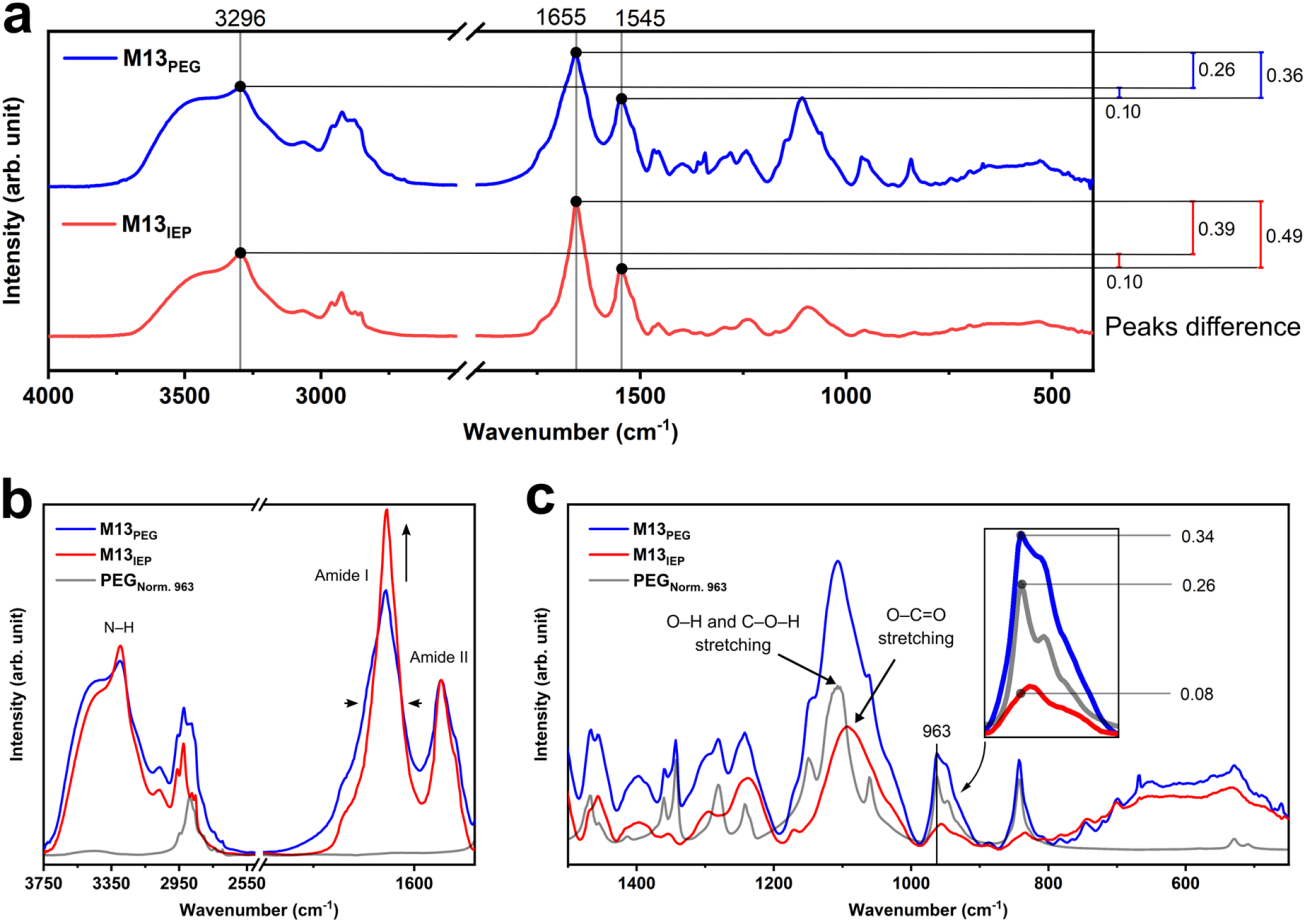
Comparison and analysis of the FT-IR spectra of M13_PEG_ and M13_IEP_. (**a**) The comparison of the FT-IR spectra of the M13_PEG_ and M13_IEP_ shows that while the amide II band maintains the same intensity ratio at the secondary amine peak at 3296 cm^−1^ after isoelectric precipitation, the amide I band shows an increased intensity. (**b**) The intensity of peaks at 1545, 1655 and 3296 cm^−1^ are not significantly affected by the presence of PEG. (**c**) The zoomed-in fingerprint range of the FT-IR spectra reveals the differences among the samples highlighting PEG’s contributions in the M13_PEG_ spectrum. M13_PEG_ and M13_IEP_ spectra were normalised relative to the intensity of the amide II band while PEG spectrum was normalised relative to the peak at 963 cm^−1^ with an intensity equal to the difference between the M13_PEG_ and the M13_IEP_ at the same wavenumber (see inset).

The comparison between the normalised spectra of the M13_PEG_ and the M13_IEP_ reveals that while the intensity ratio between the peaks at 1545 and 3296 cm^−1^ does not show variations, the intensity ratio between those and the peak at 1655 cm^−1^ is changed (Fig. 4a). This is further emphasised after normalising the spectra relative to the amide II band intensities (Fig. 4b). Given that PEG does not have absorption peaks corresponding to the above wavenumbers (1500–2500 cm^−1^ and 3000–4000 cm^−1^) and that the isoelectric precipitation is supposed to reduce the contaminant PEG, we expected no variation of the amide I and II bands, and the reduction of the intensity of the common peaks of M13 and PEG across the entire fingerprint region of the spectra (1500–400 cm^−1^) (Figs. 3, 4a). Surprisingly, the M13_IEP_ amide I band showed a 30% increment compared to the M13_PEG_, while the other characteristic peaks have decreased, as expected (Figs. 3, 4c). The amide I band is typical of M13 and it is absent in PEG. Therefore, assuming that the isoelectric precipitation was performed to selectively reduce the amount of PEG from M13_PEG_ without altering the structure of the virus, and consequently, its FT-IR spectrum, the increment of the amide I band was unexpected. However, this could be associated with the effect of the reduced PEG molecules attached to the surface of M13. We do not exclude the possibility that the presence of PEG could have been interfering with the vibrational modes of the numerous amino and carboxyl groups present on the external surface of the viral capsid^37^. Therefore, these chemical groups were able to show higher absorption values upon PEG removal via isoelectric precipitation (M13_IEP_).

Interestingly, further analysis of the fingerprint region in Fig. 4c, reveals that the intensity of the majority of the M13_PEG_ peaks correlated to the presence of PEG decreases in the M13_IEP_ spectra. It is also notable that the peaks in the range between 1000–1200 cm^−1^ change when comparing M13_PEG_ with the M13_IEP_. In particular, the spectrum of PEG normalised to the peak at 963 cm^−1^ with the intensity equal to the difference between the M13_PEG_ and M13_IEP_ at the same wavenumber (Fig. 4c), is characterised by a triplet, with the highest peak centred at 1108 cm^−1^, corresponding to O–H and C–O–H stretching. Differently, within the same range, M13_IEP_ shows a peak at 1093 cm^−1^, corresponding to the O–C=O stretching. The absence of the triplet centred at 1108 cm^−1^ suggests that PEG is either absent or its concentration is negligible in the sample M13_IEP_.

**Figure 5:**
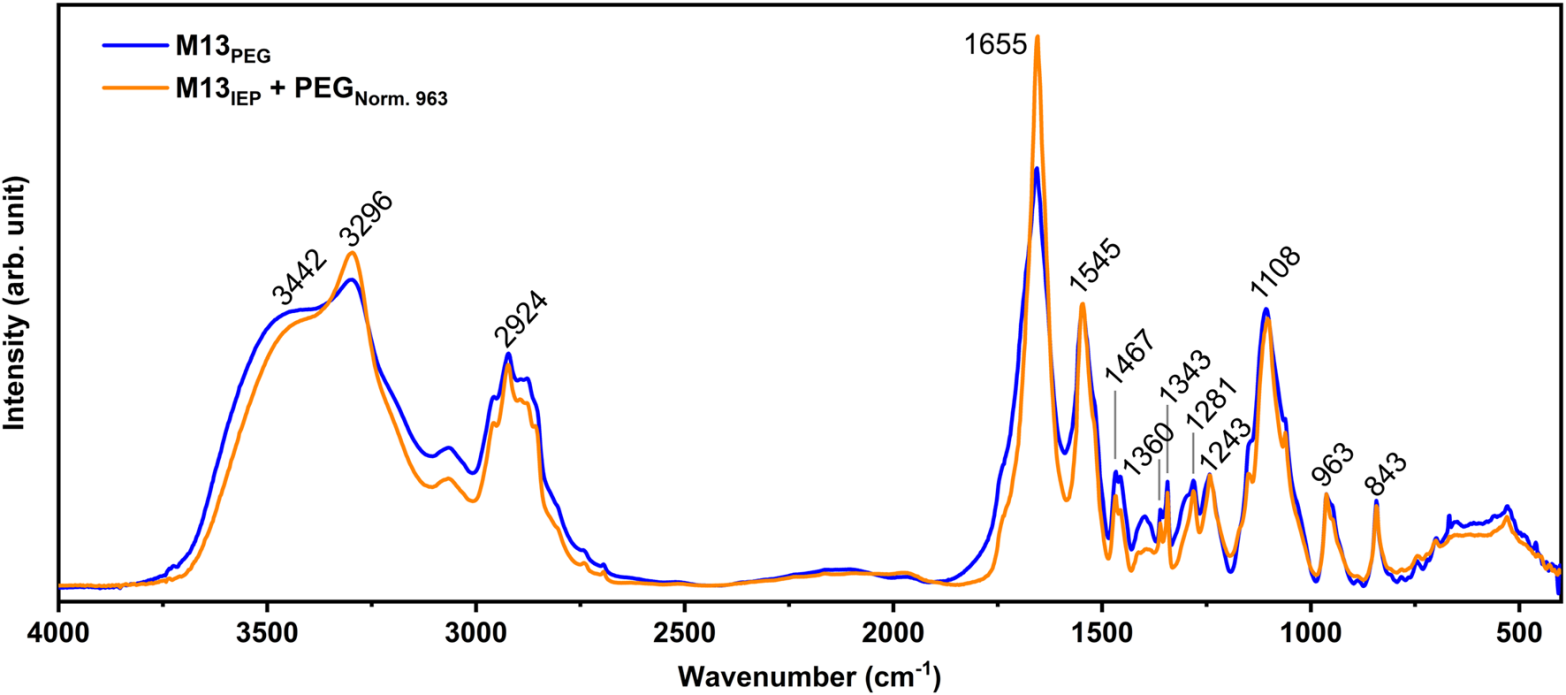
FT-IR spectra of M13_PEG_. The figure shows the comparison between the M13_PEG_ measured experimentally (blue), versus the M13_PEG_ spectra obtained via summing up the spectrum of M13_IEP_ and PEG (orange). Both M13_PEG_ spectra are normalised relative to the amide II band at 1545 cm^−1^.

Therefore, assuming that the acquired spectrum of the M13_IEP_ does not contain any traces of PEG (or very negligible amount), the sum of its signal with the signal of PEG at 953 cm^−1^ should be generating a spectrum corresponding to the acquired M13_PEG_ (Fig. 5). This PEG spectrum corresponds to the real contribution of PEG in the formation of the M13_PEG_ spectrum.

The resulting spectrum M13_PEG_ (orange) shows the majority of its peaks centred around the same wavenumber of the corresponding M13_PEG_ peaks (blue). Although the spectra show the same peak positions with similar intensities, the notable difference observed between the peaks at 1655 cm^−1^ can be justified by the fact that the calculated values of the M13_PEG_ (orange) cannot simulate the effect of PEG in altering the molecular vibrational modes corresponding to the amide band I, resulting in a theoretically higher intensity relative to the experimentally measured values.

Therefore, the analysed FT-IR spectra suggest that the isoelectric treatment is an effective method to reduce the contaminant PEG as well as highlighting FT-IR spectroscopy as a potent technique to observe the effect of the proposed purification method and for the rapid characterisation of M13 in presence of other molecules or components. Our findings highlight the significance of performing subsequent isoelectric precipitation after PEG precipitation to remove the excessive PEG on the surface of M13. This, in turn, can alter or tune the reactivity of the viral particles as well as alter the light absorption properties of the M13. This is of importance for the future potential exploitations of the M13 for the fabrication of new nanostructures and nanomaterials in the continually growing field of bio-nanotechnology.

## Conclusions

The quality of M13 stocks employed in the self-assembly of M13-based nanostructures and materials is crucial for the subsequent scalability and manufacturing of biosensors, scaffolds, batteries and other electronic components. Therefore, it is necessary to adopt optimised methodologies, which are better aligned to industrial-scale processes to achieve both the purest quality and highest possible quantity of the M13, which in turn, will improve the reactivity and the scalability of the derived M13-based nanostructures and hybrid nanomaterials.

This study demonstrates ways to improve the batch propagation and purification via PEG/isoelectric precipitation of the M13 bacteriophage, further combined with an alternative and facile approach to detect the presence of the residual PEG in the newly produced samples via the FT-IR spectroscopy. This methodology can be employed for propagation and purification of the M13 either in research laboratories or the industrial settings using bioreactors for large-scale production. Our method showed that the isoelectric precipitation causes a partial loss of sample, which corresponds to about 6% of the total amount. The FT-IR analysis revealed a drastic reduction in the PEG contaminant. We further showed that the peak ratio of the amide I and II bands (1545 and 1655 cm^−1^) is a facile and reliable way to monitor the effect of isoelectric precipitation on PEG-purified M13 stocks produced for the fabrication of novel M13-based self-assembled nanostructures and nanomaterials. Although PEG and isoelectric precipitation are two well-known techniques widely employed for the purification of M13, we have demonstrated that combining both methods leads to M13 stocks of a much higher purity. Furthermore, not only that this procedure can be scaled up, but it is also advantageous in terms of time and cost required, compared to the existing methodologies which typically involve more sophisticated and complex purification apparatuses, and which are costly and time-consuming. It is also important to mention that the effect of contaminant PEG in specific assembly conditions might be negligible due to the dispersion of M13PEG in larger reaction volumes, different pH and other specific environments. Nevertheless, our method, combined with FT-IR analysis, represents a necessary advance for the production and purification of the M13. This work will be beneficial to the wide scientific community and, the developed versatile methodology can be further applied for scaled-up production of M13 as well as adapted to the modified versions of the M13, designed for specific bio-nanoassemblies.

## Supplementary information

Supplementary Information
