# Supplementary material for: Improvements in the production of purified M13 bacteriophage bio-nanoparticle
Source: Sci Rep. 2020 Oct 29;10:18538. doi: 10.1038/s41598-020-75205-3 (PMC7596064; doi:10.1038/s41598-020-75205-3)
Supplement: Supplementary file 1 — Supplementary Information [file 41598_2020_75205_MOESM1_ESM.docx]

**Supplementary Information**

**Improvements in the production of purified**

**M13 bacteriophage bio-nanoparticle**

*Paolo Passaretti^a^, Inam Khan^b^,* *Timothy R. Dafforn^c^ and Pola Goldberg Oppenheimer^d^*

^a^ Institute of Cancer and Genomic Sciences, University of Birmingham, Birmingham, B15 2TT, UK

^b^ School of Metallurgy and Materials, University of Birmingham, Birmingham, B15 2TT, UK

^c^ School of Biosciences, University of Birmingham, Birmingham, B15 2TT, UK

^d^ School of Chemical Engineering, University of Birmingham, Birmingham, B15 2TT, UK

| **Table S1.    FT-IR peaks of PEG** | | | | |
| --- | --- | --- | --- | --- |
|  | **Wavenumber (cm^-1^)** | **Intensity (arb. unit)** | **Assignment** | **References** |
| 1 | 510 | 0.04 | - | ^1–4^ |
| 2 | 529 | 0.07 | - |  |
| 3 | 843 | 0.43 | C–C skeletal vibration |  |
| 4 | 947 | 0.30 |  |  |
| 5 | 962 | 0.45 |  |  |
| 6 | 1,060 | 0.44 | C–O stretching aliphatic ether |  |
| 7 | 1,108 | 1.00 | O–H and C–O–H stretching |  |
| 8 | 1,150 | 0.56 | C–OH stretching |  |
| 9 | 1,243 | 0.28 | C–O–C asymmetric stretching |  |
| 10 | 1,281 | 0.40 | O–H and C–O–H stretching |  |
| 11 | 1,343 | 0.55 | C–H bending |  |
| 12 | 1,360 | 0.32 | Alkyl C–H deforming |  |
| 13 | 1,414 | 0.08 | - |  |
| 14 | 1,467 | 0.33 | C–H bending |  |
| 15 | 2,693 | 0.07 | C–H stretching |  |
| 16 | 2,740 | 0.08 | - |  |
| 17 | 2,805 | 0.20 | - |  |
| 18 | 2,888 | 0.58 | Alkyl C–H stretching |  |
| 19 | 2,946 | 0.23 | - |  |
| 20 | 3,435 | 0.05 | O–H stretching |  |

| **Table S2.    FT-IR peaks of M13_PEG_** | | | | |
| --- | --- | --- | --- | --- |
|  | **Wavenumber (cm^-1^)** | **Intensity (arb. unit)** | **Assignment** | **References** |
| 1 | 529 | 0.20 | - | ^1–6^ |
| 2 | 668 | 0.18 | - |  |
| 3 | 698 | 0.13 | - |  |
| 4 | 744 | 0.08 | - |  |
| 5 | 843 | 0.20 | C–C skeletal vibration |  |
| 6 | 947 | 0.20 |  |  |
| 7 | 963 | 0.23 |  |  |
| 8 | 1,060 | 0.41 | C–O stretching aliphatic ether |  |
| 9 | 1,108 | 0.67 | O–H and C–O–H stretching |  |
| 10 | 1,150 | 0.35 | C–OH stretching |  |
| 11 | 1,171 | 0.15 | C–C and C–O–H stretching |  |
| 12 | 1,236 | 0.25 | Amide III band:  C–N stretching, N–H in-plane bending and C–H_2_ wagging |  |
| 13 | 1,243 | 0.28 | Alkyl aryl ether C–O stretching |  |
| 14 | 1,281 | 0.26 | O–H and C–O–H stretching |  |
| 15 | 1,296 | 0.23 | C−N stretching |  |
| 16 | 1,343 | 0.26 | C–H bending |  |
| 17 | 1,360 | 0.20 | Alkyl C–H deforming |  |
| 18 | 1,397 | 0.18 | Symmetric C–H_3_ bending |  |
| 19 | 1,456 | 0.27 | C–H_2_ scissoring, C–H_2_ bending and asymmetric C–H_3_ bending |  |
| 20 | 1,467 | 0.29 | C–H bending |  |
| 21 | 1,545 | 0.67 | Amide II band: amide plane N–H bending and C–N stretching |  |
| 22 | 1,656 | 1.00 | Amide I band: Amide plane C=O stretching |  |
| 23 | 2,693 | 0.07 | C–H stretching |  |
| 24 | 2,740 | 0.10 | - |  |
| 25 | 2,805 | 0.20 | O−H and N−H groups involved in hydrogen bonds |  |
| 26 | 2,856 | 0.44 |  |  |
| 27 | 2,875 | 0.49 |  |  |
| 28 | 2,893 | 0.50 | C–H stretching |  |
| 29 | 2,922 | 0.56 | O−H and N−H groups involved in hydrogen bonds |  |
| 30 | 2,957 | 0.46 |  |  |
| 31 | 3,066 | 0.34 |  |  |
| 32 | 3,296 | 0.74 |  |  |
| 33 | 3,442 | 0.66 |  |  |

| **Table S3.    FT-IR peaks of M13_IEP_** | | | | |
| --- | --- | --- | --- | --- |
|  | **Wavenumber (cm^-1^)** | **Intensity (arb. unit)** | **Assignment** | **References** |
| 1 | 531 | 0.11 | - | ^1–6^ |
| 2 | 698 | 0.08 | - |  |
| 3 | 743 | 0.05 | - |  |
| 4 | 833 | 0.03 | C–C skeletal vibration |  |
| 5 | 955 | 0.06 |  |  |
| 6 | 1,093 | 0.22 | O–C=O stretching |  |
| 7 | 1,170 | 0.04 | C–C and C–O–H stretching |  |
| 8 | 1,238 | 0.13 | Amide III band:  C–N stretching, N–H in-plane bending and C–H_2_ wagging |  |
| 9 | 1,296 | 0.07 | C−N stretching |  |
| 10 | 1,352 | 0.03 | - |  |
| 11 | 1,395 | 0.05 | Symmetric C–H_3_ bending |  |
| 12 | 1,456 | 0.09 | C–H_2_ scissoring, C–H_2_ bending and asymmetric C–H_3_ bending |  |
| 13 | 1,545 | 0.51 | Amide II band: amide plane N–H bending and C–N stretching |  |
| 14 | 1,655 | 1.00 | Amide I band: Amide plane C=O stretching |  |
| 15 | 2,854 | 0.17 | O−H and N−H groups involved in hydrogen bonds |  |
| 16 | 2,875 | 0.18 |  |  |
| 17 | 2,924 | 0.32 |  |  |
| 18 | 2,960 | 0.25 |  |  |
| 19 | 3,066 | 0.19 |  |  |
| 20 | 3,296 | 0.60 |  |  |
| 21 | 3,441 | 0.46 |  |  |

# **References**

1. Saravanan, L. & Subramanian, S. Surface Chemical Studies on the Competitive Adsorption of Poly(ethylene glycol) and Ammonium Poly(methacrylate) onto Alumina. *J. Colloid Interface Sci.* **284**, 363–377 (2005).

2. Tunç, S. & Duman, O. The Effect of Different Molecular Weight of Poly(Ethylene Glycol) on the Electrokinetic and Rheological Properties of Na-Bentonite Suspensions. *Colloids Surfaces A Physicochem. Eng. Asp.* **317**, 93–99 (2008).

3. Larkin, P. *Infrared and Raman Spectroscopy - Principles and Spectral Interpretation*. *Elsevier* (Elsevier, 2011).

4. Shameli, K. *et al.* Synthesis and Characterization of Polyethylene Glycol Mediated Silver Nanoparticles by the Green Method. *Int. J. Mol. Sci.* **13**, 6639–6650 (2012).

5. Dong, D., Zhang, Y., Sutaria, S., Konarov, A. & Chen, P. Binding Mechanism and Electrochemical Properties of M13 Phage-Sulfur Composite. *PLoS One* **8**, 1–10 (2013).

6. Katarzyna, S.-K. *et al.* Modified Filamentous Bacteriophage as a Scaffold for Carbon Nanofiber. *Bioconjug. Chem.* **27**, 2900–2910 (2016).
